# Pseudoachalasia Secondary to Metastatic Osteosarcoma

**DOI:** 10.7759/cureus.73537

**Published:** 2024-11-12

**Authors:** Isha Bansal, Yogesh Bade, Amol S Dahale, Debabrata Banerjee

**Affiliations:** 1 Medical Gastroenterology, Dr. D.Y. Patil Medical College, Hospital and Research Centre, Dr. D.Y. Patil Vidyapeeth (Deemed to be University), Pune, IND; 2 Gastroenterology, Govind Ballabh Pant Institute of Postgraduate Medical Education and Research, New Delhi, IND; 3 Gastroenterology and Hepatology, Command Hospital, Kolkata, IND

**Keywords:** dysphagia surgery, lower end of esophagus, nodal metastasis, pseudoachalasia, secondary osteosarcoma

## Abstract

Pseudoachalasia is a disorder in which symptoms, radiologic, endoscopic, and manometric results resemble idiopathic achalasia. Although these diagnoses may appear similar, their underlying causes and therapy differ significantly. Pseudoachalasia is frequently associated with malignancy, particularly primary adenocarcinoma of the esophagus or cardia. We present a 75-year-old female patient with metastatic osteosarcoma who presented with symptoms of dysphagia and typical esophageal manometry findings of achalasia. Esophagoscopy showed resistance while crossing the gastroesophageal junction. Abdominal computed tomography showed a mediastinal mass. An endoscopic ultrasound was performed, and a fine needle biopsy was taken from mediastinal deposits. Histopathology, which showed signet cell tumor cells. As the cancer was inoperable, chemotherapy was started and her symptoms have decreased from before. Typical esophageal dysmotility can be seen in pseudoachalasia, a secondary type of achalasia caused mostly by cancer or benign tumors, surgical complications, or paraneoplastic disorders. Hence we can conclude diagnosing pseudoachalasia earlier can improve outcomes.

## Introduction

Achalasia is a condition in which the lower esophageal sphincter (LES) does not fully relax, resulting in impaired oesophageal motility. There are two forms of achalasia, primary and secondary. Primary achalasia is an idiopathic motility disease. When symptoms, radiologic, endoscopic, and manometric results resemble those of idiopathic achalasia, the disease is known as pseudoachalasia. Pseudoachalasia can occur in a variety of malignant and benign pathologies. The most prevalent underlying lesion is a primary malignant tumor of the proximal stomach or distal esophagus; accounting for between 50-75% of instances with pseudoachalasia [1]. Various cancers can be worsened by the development of pseudoachalasia, either by direct invasion from neighboring structures or local mass impact from metastatic deposits. Barium esophagography offers a 25% sensitivity, whereas esophagogastroduodenoscopy has a 67% sensitivity for detecting malignancies in individuals with pseudoachalasia [2]. Early recognition of pseudoachalasia requires maintaining a high index of suspicion, particularly in patients presenting with esophageal motility issues and risk factors for malignancy.

## Case presentation

A 75-year-old female patient came to the outpatient clinic with complaints of dysphagia for the past four weeks. She experienced esophageal dysphagia, more prominent with solids than liquids, and had a history of food impaction. There were no reported incidents of odynophagia, regurgitation, or hoarseness of voice. She also reported a 7 kg weight loss over the course of four weeks, along with easy fatigability and anorexia. There was no history of abdominal pain, distension, fever, or night sweats. One year ago, the patient was diagnosed with left tibial osteosarcoma, which led to an above-knee amputation and subsequent radiotherapy. She is currently on antihypertensive and antidiabetic medications. Upon presentation, she was afebrile and vitally stable. Systemic examination revealed no abnormalities. She brought along an outside barium swallow report that suggested achalasia cardia (Rat-tail appearance). The laboratory investigations at the time of presentation were normal except for elevated glycated hemoglobin (HBA1c) (Table 1).

**Table 1:** Laboratory investigations. TLC: total leucocyte count, HBA1c: glycated hemoglobin, INR: international normalized ratio.

Investigation	Value	Normal value
Hemoglobin	13.4.1 g/dL	11.6- 15 g/dL
TLC	7400 cells/μL	4000- 10000 cells/μL
Platelets	262,000 cells/μL	150,000- 410000 cells/μL
Total bilirubin	0.53 mg/dL	0.22-1.2 mg/dL
Conjugated bilirubin	0.26 mg/dL	0.1-1.0 mg/dL
Serum glutamic oxaloacetic transaminase	7 U/L	8-43 U/L
Serum glutamate pyruvate transaminase	13 U/L	7-45 U/L
Alkaline phosphatase	85 U/L	50-117 U/L
Gamma glutamyl transferase	32 U/L	5-36 U/L
Urea	21 mg/dL	17-49 mg/dL
Creatinine	0.61 mg/dL	0.6-1.2 mg/dL
Serum albumin	4.1 g/dL	3.5 5.2 g/dL
HBA1c	9.2 gm/dL	Upto 6.4 gm/dL
Prothrombin time/INR	13.7/1.16	14/1

**Figure 1:**
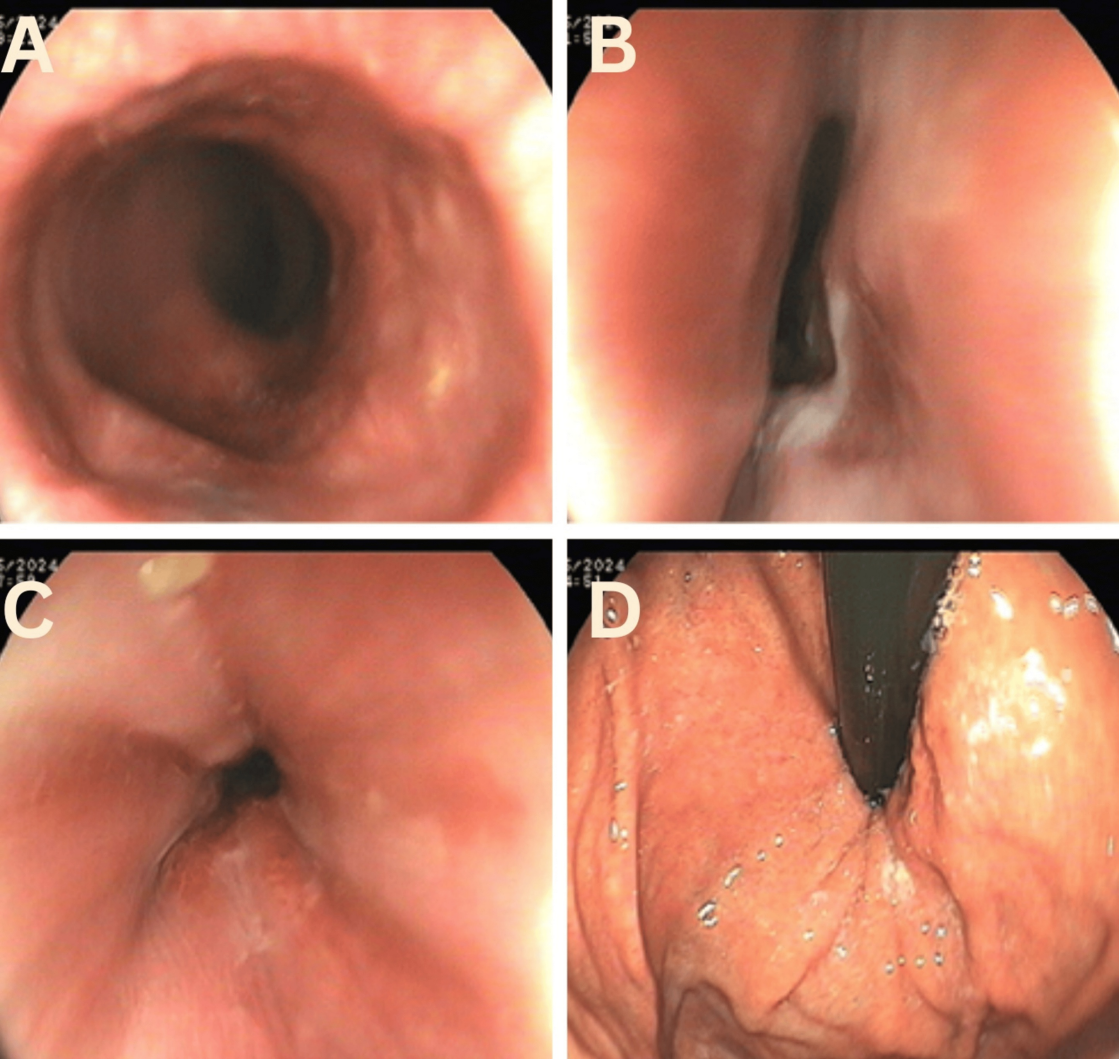
Upper esophagogastroduodenoscopy showing dilated esophagus (A), narrowing at lower esophagus (B and C). There was resistance while crossing gastroesophageal junction (D).

Esophagogastroduodenoscopy showed luminal narrowing at 34 cm from incisors; there was resistance to the passage of scope through the gastroesophageal junction (Figure 1).

**Figure 2:**
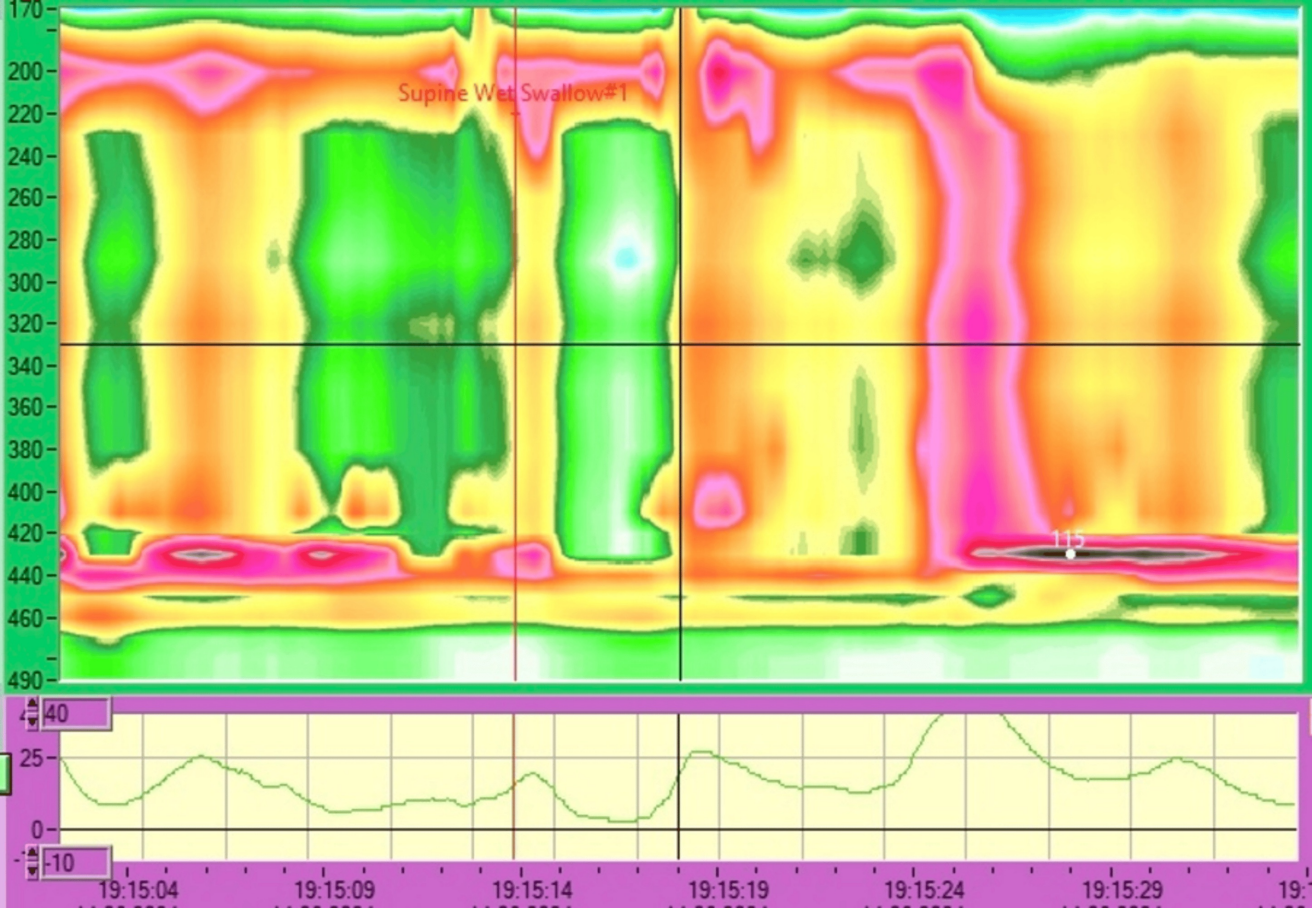
High-resolution esophageal manometry findings include raised LES pressure, decreased relaxation and raised IRP. LES: lower esophageal sphincter, IRP: intra-esophageal resting pressure.

High-resolution esophageal manometry findings included raised LES pressure, decreased relaxation, and raised intra-esophageal resting pressure (IRP), which raised suspicion of achalasia (Figure 2).

**Figure 3:**
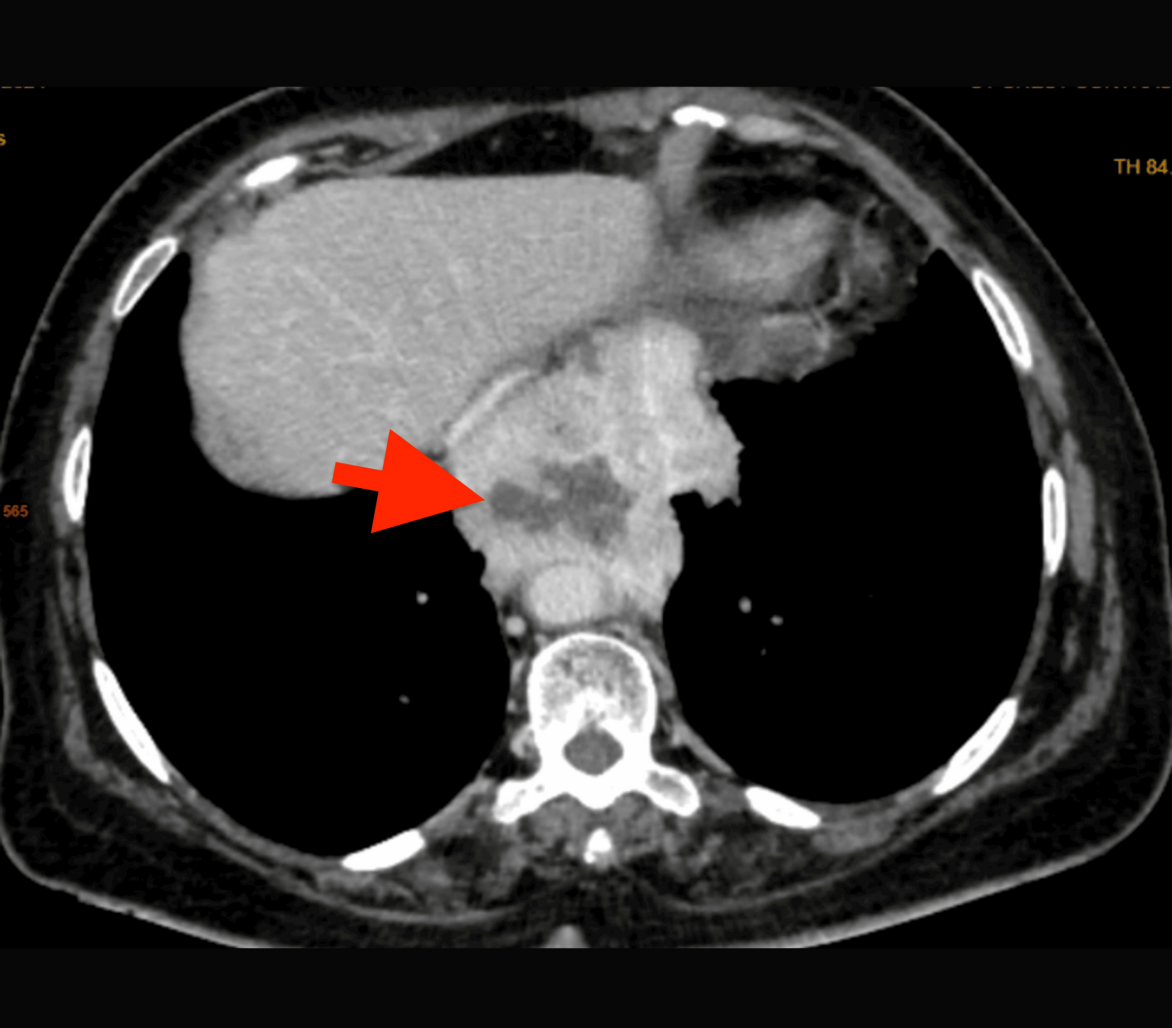
Contrast-enhanced computed tomography; mixed density lesion size 64x84 mm noted in posterior mediastinum showing patchy peripheral post-contrast enhancement with central non-enhancing areas with multiple hyperdense foci with epicenter esophagus. Red arrow: Esophagus.

The patient underwent computed tomography, subsequently showing a well-defined mixed density lesion size 64x84 mm, noted in the posterior mediastinum showing patchy peripheral post-contrast enhancement with central non-enhancing areas with multiple hyperdense foci and epicenter esophagus (Figure 3).

**Figure 4:**
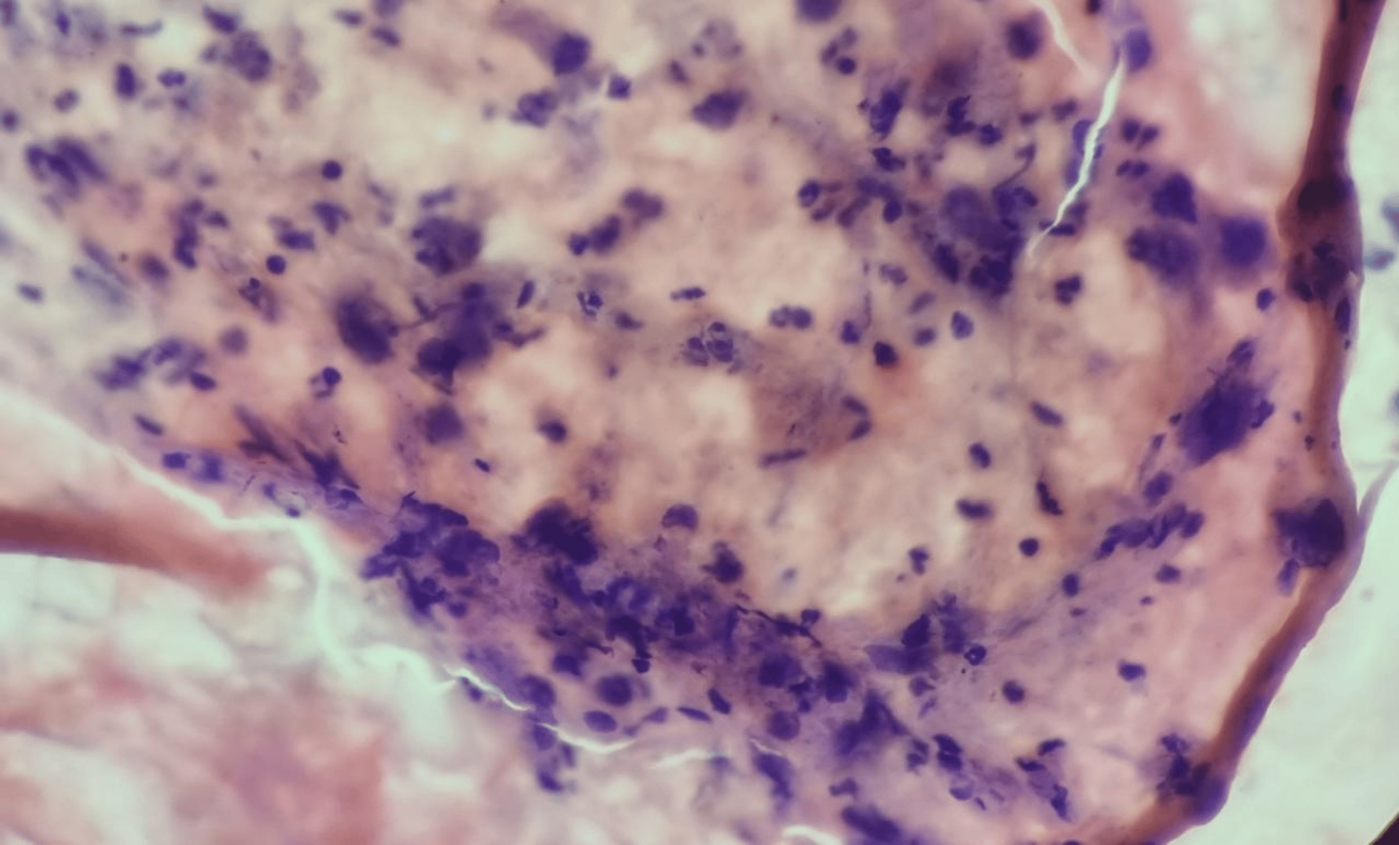
Histopathology showed sheets and clusters of round to ovoid as well as spindle shaped tumor cells.

Endosonography done of the patient showed hyperechoic thickening beyond the esophageal wall at 34 cm suggestive of mediastinal deposits; a biopsy was taken from the lesion. Biopsy taken of mediastinal deposits showed moderately cellular fine needle aspiration smears with sheets and clusters of round to ovoid as well as spindle-shaped tumor cells. These cells had moderate cytoplasm with fine nuclear chromatin-positive for malignant cells (Figure 4).

She was started on chemotherapy drugs high-dose methotrexate, doxorubicin, and cisplatin. Her symptoms subsided after receiving the second cycle of the chemotherapeutic regimen. The patient is on follow-up with a medical oncologist for further management.

## Discussion

Pseudoachalasia is an uncommon condition that affects only a tiny number of people with dysphagia. Howarth originally defined pseudoachalasia as a malignant form in 1919, followed by Ogilvie [2]. The tumor most likely to produce pseudoachalasia is esophageal or cardia carcinoma, which has classic esophagogastric junction strictures. Submucosal invasion with subsequent destruction of the myenteric plexus is less prevalent [2]. Aside from this link, pseudoachalasia has also been documented as a sign of such malignancies as pancreatic cancer, pleural mesothelioma, lung cancer, multiple myeloma, and metastatic breast and cervical carcinoma.

Early diagnosis of malignancy-associated pseudoachalasia is crucial to avoiding inappropriate treatment and delaying necessary treatments. Distinguishing between idiopathic and pseudoachalasia is challenging since both conditions commonly overlap clinical and diagnostic features, such as radiographic examinations, endoscopy, and manometry, as was the case with our patient. Pseudoachalasia is more common in the elderly (>50 years) and is characterized by sudden start of symptoms, fast progression, and weight loss [3].

For the majority of individuals with pseudoachalasia, a chest and abdomen CT scan is performed at some point throughout the diagnostic process. As an imaging method, it has low sensitivity for identifying tumors surrounding the junction, even those with extensive local invasion [4].

To the best of our knowledge, our case is the first recorded instance of primary osteosarcoma-producing pseudoachalasia, according to the literature review. This patient's secondary achalasia is supported by a lack of reaction to pneumatic dilation, strong resistance during upper endoscope passage across the gastroesophageal junction, radiographic abnormalities, and fast worsening of symptoms. We identified the mass before diagnosing achalasia, but it is important to be vigilant of pseudoachalasia in individuals who fulfill achalasia criteria but do not respond to traditional therapy [3].

The pathogenesis of this disorder is still far from being clearly understood: the alterations in esophageal motor function are believed to be a consequence of several possible mechanisms: mechanical compression of the esophageal wall, infiltration of the muscle layers, and autoimmune damage to the myenteric plexus [5]. Primary malignancy of the esophagogastric junction accounts for 54% to 70% of pseudoachalasia cases, with adenocarcinomas of the gastric fundus or distal esophagus being the most commonly implicated tumors [6]. While many forms of malignancy can metastasize to the esophagus, only 6% of pseudoachalasia cases are secondary to metastatic versus primary disease [7]. A recent study showed that distal esophageal wall thickening that is nodular or lobulated and asymmetric; a soft-tissue mass at the esophagogastric junction; mediastinal lymphadenopathy; and pulmonary, hepatic, or osseous metastases are findings that point to secondary achalasia [8].

## Conclusions

Pseudoachalasia should be suspected in elderly patients with a rapid onset of symptoms, significant weight loss, and difficulty passing the esophagogastric junction during endoscopy, especially when typical treatments fail to provide relief. In such cases, pseudoachalasia may be an early indication of an underlying malignancy. Since both idiopathic and pseudoachalasia share similar clinical and diagnostic features, distinguishing between the two can be particularly challenging. However, timely recognition of malignancy-related pseudoachalasia is crucial to avoid inappropriate management and ensure that patients receive prompt and appropriate therapeutic interventions.
